# Supplementary material for: Dreaming with hippocampal damage
Source: eLife. 2020 Jun 8;9:e56211. doi: 10.7554/eLife.56211 (PMC7279885; doi:10.7554/eLife.56211)
Supplement: Supplementary file 1. — This table provides details of the neuropsychological profile (summary data and statistical analyses) of the patients across a range of cognitive tests, and indicates the selective nature of their memory loss. [file elife-56211-supp1.docx]

**Supplementary File 1. Summary of neuropsychological information.**

|  |  |  |  |  |  |  |  |
| --- | --- | --- | --- | --- | --- | --- | --- |
|  | HPC |  | CTL^a^ |  |  |  |  |
|  | M (SD) |  | M (SD) |  | U | ES | P-Value |
| *General cognition* |  |  |  |  |  |  |  |
| WASI Similarities scaled score^1^ | 11.75 (0.96) |  | 11.83 (2.55) |  | 21 | 0.18 | 0.713 |
| *Episodic memory* |  |  |  |  |  |  |  |
| Autobiographical Interview^2^ internal (‘episodic’) details^b^ | 32.55 (5.63) |  | 46.43 (12.82) |  | 3.0 | 1.65 | **0.030** |
| Autobiographical Interview total word count^b^ | 7266.75 (2918.23) |  | 5224.13 (2513.48) |  | 10.0 | 0.62 | 0.308 |
| WMS Logical Memory^3^ (immediate recall, units) | 8.00 (2.71) |  | 12.58 (3.18) |  | 7.0 | 1.20 | **0.038** |
| WMS Logical Memory (immediate recall, thematic) | 8.00 (2.16) |  | 13.75 (2.96) |  | 3.0 | 1.65 | **0.010** |
| WMS Logical Memory (delayed recall, units) | 7.25 (2.87) |  | 13.17 (3.74) |  | 4.0 | 1.53 | **0.015** |
| WMS Logical Memory (delayed recall, thematic) | 7.25 (3.10) |  | 13.50 (3.18) |  | 3.5 | 1.59 | **0.012** |
| WMS Word Lists (immediate recall) scaled score | 9.50 (3.87) |  | 13.25 (3.17) |  | 11.5 | 0.82 | 0.126 |
| WMS Word Lists (delayed recognition) scaled score | 10.00 (2.71) |  | 11.67 (1.37) |  | 17.0 | 0.43 | 0.373 |
| Rey-Osterrieth Complex Figure^4^ (immediate recall /36) | 19.50 (6.61) |  | 23.8 (7.36) |  | 10.5 | 0.90 | 0.178 |
| Rey-Osterrieth Complex Figure (delayed recall, /36) | 19.75 (5.06) |  | 23.92 (7.75) |  | 11.0 | 0.86 | 0.113 |
| Warrington Recognition Memory Test^5^ for Words scaled score | 11.25 (0.96) |  | 12.00 (2.34) |  | 18.5 | 0.34 | 0.495 |
| Warrington Recognition Memory Test for Faces scaled score | 8.00 (2.16) |  | 11.33 (2.96) |  | 7.0 | 1.20 | **0.036** |
| *Semantic memory* |  |  |  |  |  |  |  |
| Warrington Graded Naming Test^6,7^ scaled score | 11.00 (2.16) |  | 13.67 (2.42) |  | 9.0 | 1.02 | 0.065 |
| Autobiographical Interview external (‘semantic’) details^b^ | 7.63 (3.74) |  | 5.90 (3.67) |  | 14.0 | 0.20 | 0.730 |
| *Attention/working memory* |  |  |  |  |  |  |  |
| WMS Digit Span (forward) scaled score | 11.50 (3.11) |  | 13.25 (3.22) |  | 19.5 | 0.28 | 0.581 |
| *Executive functions* |  |  |  |  |  |  |  |
| D-KEFS Letter Fluency Test (FAS)^8^ scaled score | 12.25 (2.87) |  | 14.33 (3.20) |  | 14.5 | 0.60 | 0.247 |
| D-KEFS Category Fluency Test scaled score | 13.50 (5.74) |  | 13.92 (4.38) |  | 22.0 | 0.12 | 0.807 |
| D-KEFS Category Switching Test scaled score | 13.00 (4.24) |  | 12.67 (2.93) |  | 22.0 | 0.12 | 0.806 |
| D-KEFS Color-Word Interference Test scaled score | 13.25 (2.50) |  | 12.42 (2.23) |  | 18.5 | 0.34 | 0.501 |
| D-KEFS Trails Test (visual scanning) scaled score | 11.25 (0.96) |  | 12.00 (1.28) |  | 15.0 | 0.57 | 0.261 |
| D-KEFS Trails Test (number sequencing) scaled score | 11.00 (1.41) |  | 11.83 (2.52) |  | 15.5 | 0.53 | 0.292 |
| D-KEFS Trails Test (letter sequencing) scaled score | 10.50 (3.00) |  | 12.50 (1.51) |  | 15.5 | 0.53 | 0.280 |
| D-KEFS Trails Test (number-letter sequencing) scaled score | 9.75 (2.06) |  | 12.83 (1.03) |  | 3.0 | 1.65 | **0.008** |
| D-KEFS Trails Test (motor speed) scaled score | 12.00 (0.82) |  | 11.75 (1.14) |  | 19.5 | 0.28 | 0.568 |
| Hayling Sentence Completion Test^9^ (coherent) | 5.75 (0.50) |  | 6.08 (1.00) |  | 20.0 | 0.24 | 0.606 |
| Hayling Sentence Completion Test (incoherent) | 5.75 (0.50) |  | 5.75 (0.75) |  | 23.0 | 0.06 | 0.882 |
| Hayling Sentence Completion Test (errors) | 6.25 (2.22) |  | 6.83 (1.11) |  | 22.0 | 0.12 | 0.800 |
| Hayling Sentence Completion Test (total) | 17.75 (2.50) |  | 18.67 (1.56) |  | 22.5 | 0.09 | 0.851 |
| *Visual perception* |  |  |  |  |  |  |  |
| VOSP^10,11^ Dot Counting (/10) | 9.50 (1.00) |  | 10.00 (0.00) |  | 18.0 | 0.37 | 0.083 |
| VOSP Position Discrimination (/20) | 19.50 (1.00) |  | 20.00 (0.00) |  | 18.0 | 0.37 | 0.083 |
| VOSP Cube Analysis (/10) | 9.50 (1.00) |  | 9.58 (0.79) |  | 23.5 | 0.03 | 0.936 |
| VOSP Overall (/40) | 38.50 (3.00) |  | 39.58 (0.79) |  | 22.5 | 0.09 | 0.811 |
| *Mood* |  |  |  |  |  |  |  |
| HADS^12^ Anxiety | 2.75 (2.06) |  | 4.33 (2.93) |  | 15.0 | 0.57 | 0.269 |
| HADS Depression | 1.25 (0.50) |  | 2.33 (2.74) |  | 22.5 | 0.09 | 0.852 |

M = mean; SD = standard deviation; ES = effect size; HPC = hippocampal-damaged patients; CTL = control participants. P-values relate to between-group non-parametric Mann-Whitney U tests with significant differences depicted in bold. WASI = Wechsler Abbreviated Scale of Intelligence; WMS = Wechsler Memory Scale III; D-KEFS = Delis-Kaplan Executive Function System; VOSP = Visual Object and Space Perception Battery; HADS = Hospital Anxiety and Depression Scale. For the Autobiographical Interview, the scores are the average number of internal (or external) details over five memories. ^a^The control group consisted of twelve participants (all males, mean age 57.2 years ± 16.6). ^b^The control group consisted of eight participants (six males, mean age 54.63 years ± 16.32).

^1-12^Denote these test materials:

1. Wechsler D. 1999. *Wechsler Abbreviated Scale of Intelligence*. New York, NY: The Psychological Corporation, Harcourt Brace.

2. Levine B, Svoboda E, Hay JF, Winocur G, Moscovitch M. 2002. Aging and autobiographical memory: Dissociating episodic from semantic retrieval. *Psychology and Aging* **17**:677–689.

3. Wechsler D. 1997. T*he Wechsler Memory Scale*, Third Edition. San Antonio, TX: The Psychological Corporation.

4. Osterrieth PA. 1944. The test of copying a complex figure: a contribution to the study of perception and memory. *Archive of Psychology* **30**:286 –356.

5. Warrington EK. 1984. *Recognition Memory Test*: Manual. Berkshire, UK: NFER-Nelson.

6. McKenna P, Warrington EK. 1980. Testing for nominal dysphasia. *Journal of Neurology, Neurosurgery and Psychiatry* **43**:781–788.

7. Warrington EK. 2010. The graded naming test: a restandardisation. *Neuropsychological Rehabilitation* **7**:143–146.

8. Delis DC, Kaplan E, Kramer JH. 2001. *Delis Kaplan Executive Function System* (D-KEFS). San Antonio, TX: The Psychological Corporation.

9. Burgess P, Shallice T. 1997. *The Hayling and Brixton Tests*. Test Manual. Bury St Edmunds, UK: Thames Valley Test Company.

10. Warrington EK, James M. 1991. A new test of object decision: 2D silhouettes featuring a minimal view. *Cortex* **27**:370 –383.

11. Gabrovska V, Laws K, McKenna P. 1996. Visual form perception in schizophrenia: further evidence for a disorder of semantic memory. *European. Psychiatry* **11**:278.

12. Zigmond AS, Snaith RP. 1983. The hospital anxiety and depression scale. *Acta Psychiatrica Scandinavia* **67**:361–370.
