# Supplementary material for: Dreaming with hippocampal damage
Source: eLife. 2020 Jun 8;9:e56211. doi: 10.7554/eLife.56211 (PMC7279885; doi:10.7554/eLife.56211)
Supplement: Supplementary file 2. — This table provides details (summary data and statistical analyses) of subjective measures of the general quality and pattern of sleep, as well as on objective measures of sleep-related breathing disorders, and sleep-wake patterns across one week. There were no significant differences between the patient and control participants on any measure. [file elife-56211-supp2.docx]

**Supplementary File 2. Sleep quality of the patients and control participants.**

|  |  |  |  |  |  |  |  |
| --- | --- | --- | --- | --- | --- | --- | --- |
|  | HPC |  | CTL |  |  |  |  |
|  | M (SD) |  | M (SD) |  | U | ES | P-Value |
| *Sleep questionnaires* |  |  |  |  |  |  |  |
| PSQI | 3.50 (1.00) |  | 4.90 (3.31) |  | 16.5 | 0.27 | 0.604 |
| ESS | 9.25 (8.34) |  | 5.00 (3.59) |  | 13.5 | 0.51 | 0.350 |
| MEQ | 53.50 (5.8) |  | 58.60 (6.06) |  | 11.5 | 0.68 | 0.228 |
| *WatchPAT* |  |  |  |  |  |  |  |
| Apnoea–Hypopnoea Index (AHI) | 17.58 (15.21) |  | 10.13 (6.66) |  | 13.0 | 0.55 | 0.322 |
| *Actigraphy (across 7 nights)* |  |  |  |  |  |  |  |
| Sleep efficiency (%) | 83.44 (9.43) |  | 89.25 (2.83) |  | 10.0 | 0.82 | 0.157 |
| Total sleep time (minutes) | 413.79 (88.33) |  | 420.49 (32.97) |  | 17.0 | 0.23 | 0.671 |
| Fragmentation index | 28.50 (13.01) |  | 18.48 (6.23) |  | 14.0 | 0.47 | 0.396 |
| Night-to-night variability | 9.82 (4.90) |  | 7.42 (2.36) |  | 15.0 | 0.39 | 0.480 |
| Bedtime | 23:50 (01:00) |  | 23:30 (00:32) |  | 18.0 | 0.15 | 0.777 |
| Midpoint | 3:41 (0:35) |  | 3:17 (0:33) |  | 11.0 | 0.72 | 0.203 |

M = mean; SD = standard deviation; ES = effect size; HPC = hippocampal-damaged patients; CTL = control participants; PSQI = Pittsburgh Sleep Quality Index; ESS = Epworth Sleepiness Scale; MEQ = Morningness-Eveningness Questionnaire. P-values relate to between-group non-parametric Mann-Whitney U tests.
